# Field-cycling imaging yields repeatable brain R_1_ dispersion measurement at fields strengths below 0.2 Tesla with optimal fitting routine

**DOI:** 10.1007/s10334-025-01230-w

**Published:** 2025-02-15

**Authors:** Nicholas Senn, P. James Ross, Reina Ayde, Vasiliki Mallikourti, Adarsh Krishna, Charly James, Clarisse F. de Vries, Lionel M. Broche, Gordon D. Waiter, Mary Joan MacLeod

**Affiliations:** 1 Aberdeen Biomedical Imaging Centre, School of Medicine, Medical Sciences and Nutrition, University of Aberdeen, Aberdeen, UK; 2 Institute of Medical Sciences, School of Medicine, Medical Sciences and Nutrition, University of Aberdeen, Aberdeen, UK; 3 AMT Center, School of Medicine, Medical Sciences and Nutrition, University of Aberdeen, Aberdeen, UK; 4 School of Health & Wellbeing, University of Glasgow, Glasgow, UK

**Keywords:** Magnetic resonance imaging, Cerebral small vessel diseases, Neuroimaging, Field-cycling imaging

## Abstract

**Objectives:**

By rapidly changing magnetic field strength between 0.2 and 200 mT during the pulse sequence Field-Cycling Imaging (FCI) makes it possible to identify and evaluate new quantitative markers of pathology derived from dispersion of spin–lattice relaxation rate (*R*_1_) in vivo. The aim of this work was to determine the most effective approach to reliably estimate multi-field *R*_1_ dispersion measurements in brain tissue using FCI.

**Materials and methods:**

This repeatability study consisted of twenty participants with moderate or severe small vessel disease. Each participant underwent 3 T MRI and FCI scans, repeated 30 days apart. After *R*_1_ maps were generated at 0.2, 2, 20, and 200 mT, co-registered tissue labels generated from 3 T MRI were used to extract tissue averaged values of *R*_1_ dispersion from regions of white matter (WM) and WM hyperintensities (WMHs).

**Results:**

The fitted model which yielded best overall image contrast between WM and WMH regions and *R*_1_ dispersion model adherence was determined. Tissue averaged values of *R*_1_ (0.2 mT) and *R*_1_ dispersion slope exhibited Cohen’s d effect sizes of 3.07 and 1.48, respectively, between regions of WM and WMH. The cohort study results were repeatable between study visits.

**Discussion:**

Differences in *R*_1_ measurements could repeatably be discerned between normal and abnormal appearing brain tissues.

**Supplementary Information:**

The online version contains supplementary material available at 10.1007/s10334-025-01230-w.

## Introduction

The way in which spin–lattice relaxation time (*T*_1_) of water protons varies (disperses) with magnetic field strength is sensitive to differences in tissue type and pathology. These *T*_1_ variations are closely linked to the frequency distribution of the thermal energy responsible for the Brownian motion of molecules that generate MRI signals [1, 2]. Therefore, the profile of the *T*_1_ dispersion can quantify the extent of molecular motion [1, 2]. Early research in MRI has shown that the profile of *T*_1_ dispersion is most sensitive to tissue structure at fields below 0.1 T [3–5]. This is because biological structures tend to have physiologically relevant dynamics at timescales longer than a microsecond, which corresponds to Larmor frequencies in the MHz range [3–5]. To realise the goal of clinically valuable quantified measurements of *T*_1_ dispersion, new ways of accessing and interpreting measurements of low-field *T*_1_ dispersion from different tissues and pathologies in vivo are required [6, 7].

By rapidly changing the magnetic field strength between 0.2 mT and 200 mT during the pulse sequence, Field-Cycling Imaging (FCI) is now making it possible to identify and evaluate new quantitative markers of pathology derived from the dispersion of spin–lattice relaxation rate (*R*_1_ = 1/*T*_1_) in patients [8]. This opens new research avenues to explore quantitative tissue contrast mechanisms at low magnetic fields that originate from pathology-dependent tissue remodelling, and to extract biomarkers from the *R*_1_ dispersion profile optimised for specific pathologies. In addition, FCI can inform on the utility of *R*_1_ mapping for different pathologies at field strengths below 0.2 T.

Our group has recently reported the discriminatory potential of *R*_1_ dispersion measures to differentiate ischaemic stroke and healthy tissue from FCI scans obtained in vivo between 0.2 mT and 200 mT [9]. However, because FCI is a novel imaging technology, only a small number of human imaging data sets have been available to evaluate the performance of analysis approaches aimed at improving the robustness of *R*_1_ measurements [10]. Moreover, the repeatability of FCI measurements in the brain has yet to be determined.

*R*_1_ values are extracted using models derived from spin–lattice recovery to fit FCI data acquired at multiple evolution magnetic field strengths and evolution times. Quantitative maps of *R*_1_ dispersion are then generated from the resulting profile of *R*_1_ values. Because of the inherently low signal-to-noise ratio available at lower field strengths, different processing approaches are needed to reduce error propagation from the acquired signal noise to *R*_1_ measures extracted from FCI data. Additional free parameters can be added to the fitting models to account for sequence-specific features like field ramp inefficiencies [10, 11]. Instead of fitting data acquired from each field separately, a multi-field fitting approach can also be used to fit imaging data acquired across all fields and evolution times simultaneously, increasing the number of data points available to fit shared model parameters [10, 12]. However, the choice of fitting model must carefully be considered, as increasing the complexity of the fitting model risks over fitting the data and reducing the sensitivity, specificity, and accuracy of the targeted outcome measures.

The primary aim of this work was to determine the most effective approach to reliably estimate *R*_1_ dispersion in brain tissue from FCI. The secondary aim was to evaluate the tissue contrast and repeatability of *R*_1_ dispersion measurements averaged from regions of white matter, grey matter, and white matter hyperintensities. This work is part of a larger study investigating the feasibility of Field-Cycling Imaging and the potential of *R*_1_ dispersion measures to discriminate between ischaemic stroke, haemorrhagic stroke, and small vessel disease, which is currently ongoing.

## Methods

As part of a larger, ethically approved (NHS Research Ethics Committee UK no. 21/NS/0128), analyses were performed on a complete cohort of 20 participants (M: 14, F: 6, aged 74.0 ± 6.0, 63–85 years) with clinically determined moderate or severe cerebral small vessel disease (SVD) [13]. Participants attended two visits 30 days apart (mean: 32 ± 7.8, 21–56 days) and underwent a 3 T MRI (Philips 3 T Achieva dStream) and a separate FCI scan on each visit. First, the impact of employing different model fitting techniques on *R*_1_ tissue contrast was evaluated in regions of white matter hyperintensities (WMHs) of presumed vascular origin as a proxy for SVD [13]. Second, using the best analysis approach, repeatability and magnitude of *R*_1_ contrast (effect size) were determined.

### Image acquisition

**Fig. 1:**
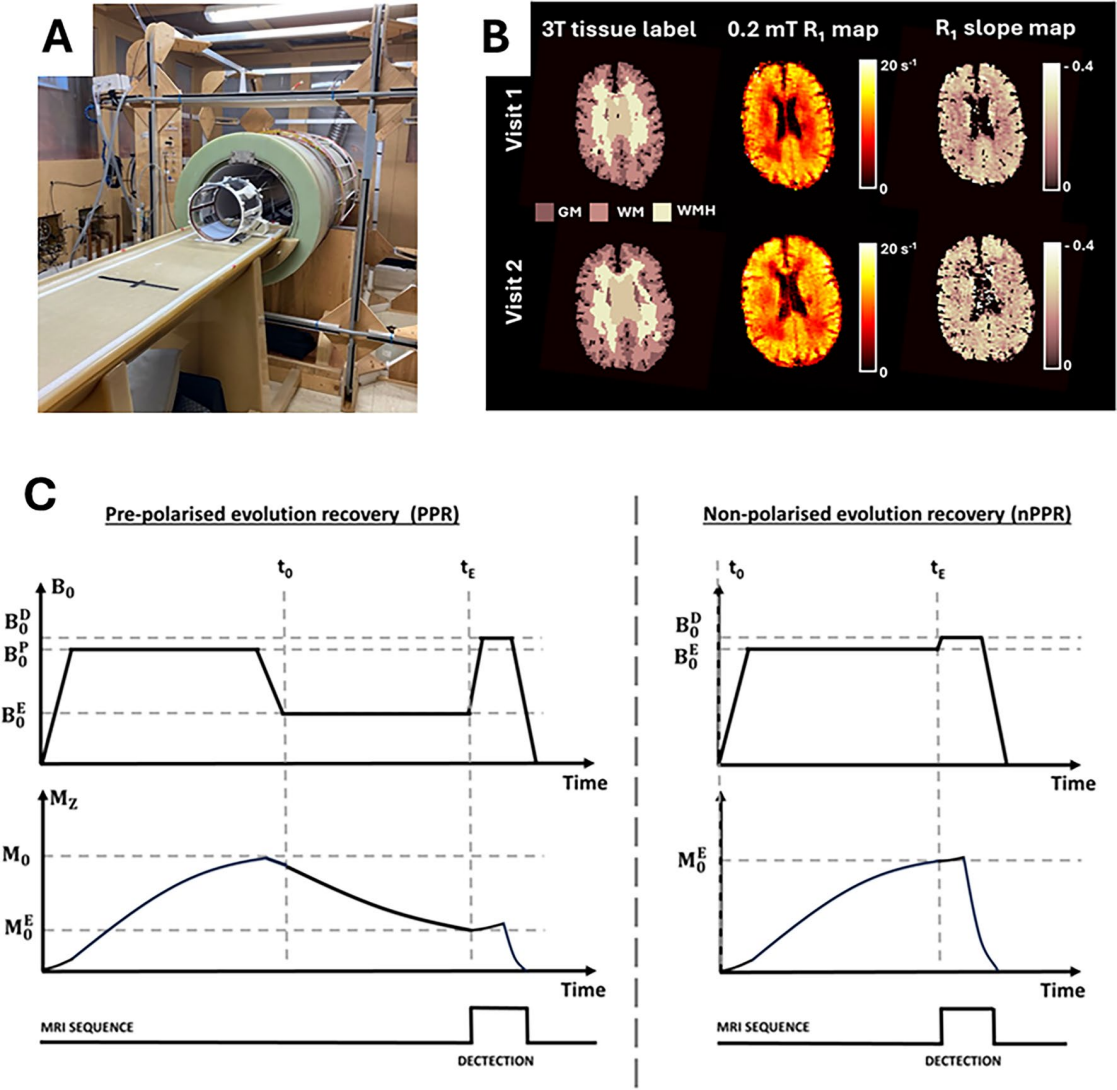
Field-Cycling Imaging (FCI) experiment. **A** Mark II FCI scanner configured for brain imaging. **B** 3T tissue label map co-registered to FCI space. Regions of white matter (WM), grey matter (GM), and white matter hyperintensity (WMH) are delineated. *R*_1_ map at evolution field strength of 0.2 mT and dispersion slope maps are shown for visits 1 and 2. **C** Pulse sequence diagram. FCI acquires images at multiple magnetic evolution field strengths and evolution times. Pre-polarised evolution recovery: to maximise available magnetisation (${\text{M}}_{\text{z}})$ prior to the evolution phase for evolution field strengths ≤ 100 mT a pre-polarisation phase is completed at field strength ${\text{B}}_{0}^{\text{p}}$, (200 mT in this study). After time point ${\text{t}}_{0}$, ${\text{M}}_{\text{z}}$ relaxes at a given evolution field (${\text{B}}_{0}^{\text{E}}$) for a specified evolution time (${\text{t}}_{\text{E}}$). ${\text{M}}_{\text{z}}({\text{t}}_{\text{E}})$ is then detected at detection field ${\text{B}}_{0}^{\text{D}}.$ ${\text{M}}_{0}$ and ${\text{M}}_{0}^{\text{E}}$ represent the equilibrium magnetisation for ${\text{B}}_{0}^{\text{p}}$ and ${\text{B}}_{0}^{\text{E}}$, respectively. Non-polarised evolution recovery: Typically for fields > 100 mT no pre-polarisation phase is needed as sufficient magnetisation will be generated from the evolution stage alone to yield adequate signal-to-noise ratio. The main magnetic field is disabled for approximately one second between shots to prevent overheating of the resistive magnet amplifiers. Therefore, no residual transverse or longitudinal magnetisation persists from shot to shot

The general FCI technique has previously been reported [10]. A brief description is provided in Fig. 1 to describe the specific approach used in this study. Field-cycling images were acquired across four evolution magnetic field strengths ($${B}_{0}^{E})$$ of 0.2, 2, 20, and 200 mT. Using the mark II FCI scanner, evolution field strengths were applied for five different evolution times, with evolution times spaced across a logarithmic scale to cover the expected range of brain tissue *R*_1_ values. These 20 images were acquired for a single image slice, with an echo time (TE) of 16 ms, matrix size of 90 × 90, and slice thickness of 10 mm. An in-plane resolution of 3.1 × 3.1 mm^2^ and 2.8 × 2.8 mm^2^ was obtained for 16 and 24 scans, respectively. The total acquisition time, to collect all 20 FCI contrasts of a single brain image slice, was 30 min. FCI data were processed to remove ghosting artefacts caused by instability in the main magnet current supply [14]. Two participants were recalled after their first scan (one after 7 days; the other after 10 days) to repeat their FCI scans, due to scanner hardware failure.

3 T MRI data were collected using a 3 T Philips Achieva dStream scanner (Best, NL), using a 32-channel head coil. *T*_1_-weighted images were acquired with repetition time (TR) of 8.1 ms, TE of 3.7 ms, acquired matrix size of 256 × 240, acquired voxel size of 1 × 1 × 1 mm^3^, and reconstructed voxel size of 0.67 × 0.67 × 1  mm^3^. Fluid attenuation inversion recovery (FLAIR) images were acquired with TR of 4800 ms, inversion time (TI) of 1650 ms, TE of 340 ms, acquired matrix size of 224 × 224, acquired voxel size of 1.12 × 1.12 × 1.12 mm^3^, and reconstructed voxel size of 0.63 × 0.63 × 0.63 mm^3^.

### Generation of 3 T MRI tissue label maps

Tissue label maps of white matter (WM), grey matter (GM), and white matter hyperintensities (WMHs) were generated from the T1W and FLAIR images obtained at 3 T MRI using standard automated approaches [15]. The FLAIR images and tissue labels were then co-registered to images obtained from FCI using a two-step manual approach in 3D Slicer 5.6.1 [16] and in-house scripts written in MATLAB (R2023a, The MathWorks, USA). First, landmarks placed across multiple slices of the FLAIR image volume and the mean FCI single slice image were used to inform a rigid-body translation so that the orientation of the FLAIR and tissue label image volumes matched the orientation of the FCI image. The location of the FLAIR image slice that matched most closely the FCI image was then used to inform the down sampling of the 3T images to match the FCI image slice thickness. Subsequently, a landmark informed non-linear deformation was performed to match the geometry of the down sampled 3T single slice images to the FCI image.

### Comparison of FCI fitting models

Prior to voxel wise fitting of acquired FCI imaging data, images were motion corrected and denoised. Motion correction was performed using a rigid-body spatial transformation to the mean FCI image using SPM12 [17]. After motion correction, images were denoised using a pretrained denoising convolutional neural network (dnCNN) approach contained within MATLAB, introduced in R2017b [18]. The supplementary materials available online contain further details of the denoising, and motion correction approaches used.

The most effective model fitting approach to inform reliable quantification of multi-field *R*_1_ measurements in brain from FCI was investigated. *R*_1_ maps were generated at each evolution field by fitting models with increasing complexity to the signal vs evolution time data in each voxel, from which a value of *R*_1_ is assigned to each voxel for every field (see Table 1). The base model describing the evolution of the signal during the field-cycling experiment was derived from Maxwell’s equations separately for when the pre-polarisation field was used (field strengths of 0.2, 2, 20 mT) and not used (200 mT) [10]. Model F1 was used to fit the data acquired at each field independently, and data acquired from multiple fields were fitted simultaneously using four different models (S1–S4). First, because the suitability of simultaneously fitting data from all fields was unknown, data acquired with pre-polarisation at 0.2, 2, and 20 mT were fitted simultaneously, and data acquired at 200 mT were fitted separately (Model S1). Second, data acquired from all fields were fitted simultaneously (Model S2). Then, a Rician noise term was added to the base model in an attempt to more accurately model the noise floor in the acquired data (Model S3). An additional weighting parameter, *β*, was added to Model S4 as an estimated correction to the magnetisation recovery occurring during both the ramp between evolution and detection fields, and the exposure to readout field before detection. Subsequently, to quantify the field dependence of the *R*_1_ measurements, quantitative maps of the *R*_1_ dispersion slope, b, were generated by fitting the power law dispersion model to the multi-field *R*_1_ values on a voxel-by-voxel basis by *R*_1_ = a(${B}_{0}^{E}$)^b^.

**Table 1:** Multi-field FCI fitting models

Model^a^	Evolution fields: 0.2, 2, 20 mT^b^	Evolution fields: 200 mT^c^	# fit parameters^d^
F1	${S}_{PPR}=\chi \left(\left(\alpha {B}_{0}^{P}- {B}_{0}^{E}\right){e}^{-{t}_{E} {R}_{1}^{E}}+ {B}_{0}^{E}\right)$	${S}_{\text{nPPR}}=\chi \left({B}_{0}^{E}\left(1-{e}^{-{t}_{E} {R}_{1}^{E}}\right)\right)$	6/20
S1	${S}_{PPR}=\chi \left(\left(\alpha {B}_{0}^{P}- {B}_{0}^{E}\right){e}^{-{t}_{E} {R}_{1}^{E}}+ {B}_{0}^{E}\right)$		5/15
S2	${S}_{PPR}=\chi \left(\left(\alpha {B}_{0}^{P}- {B}_{0}^{E}\right){e}^{-{t}_{E} {R}_{1}^{E}}+ {B}_{0}^{E}\right)$	${S}_{\text{nPPR}}=\chi \left({B}_{0}^{E}\left(1-{e}^{-{t}_{E} {R}_{1}^{E}}\right)\right)$	6/20
S3	${S}_{PPR}=\chi \left(\left(\alpha {B}_{0}^{P}- {B}_{0}^{E}\right){e}^{-{t}_{E} {R}_{1}^{E}}+ {B}_{0}^{E}\right)$	${S}_{\text{nPPR}}=\chi \left({B}_{0}^{E}\left(1-{e}^{-{t}_{E} {R}_{1}^{E}}\right)\right)$+ C	7/20
S4	${S}_{PPR}=\chi \left(\left(\alpha {B}_{0}^{P}- {B}_{0}^{E}\right){e}^{-{t}_{E} {R}_{1}^{E}}+ {B}_{0}^{E}+ \beta \right)$	${S}_{\text{nPPR}}=\chi \left({B}_{0}^{E}\left(1-{e}^{-{t}_{E} {R}_{1}^{E}}\right)+ \beta \right)$	7/20

^a^F1, fitting models were applied separately to data acquired at each evolution field. S1, the fitting model was fitted simultaneously for data acquired from 0.2, 2, 20 mT only; data acquired at 200 mT was unused. S2–S4, data acquired from all fields were fitted simultaneously

^b^Models fit to data acquired at evolution fields 0.2, 2, and 20 mT. ${\text{B}}_{0}^{\text{E}}$, evolution field. ${\text{B}}_{0}^{\text{P}}$, polarisation field. ${\text{R}}_{1}^{\text{E}}$, relaxation rate at each evolution field. $\upchi ,$ proportionality constant. ${\text{S}}_{\text{PPR}}$, acquired magnitude signal in cases of pre-polarisation field applied. ${\text{S}}_{\text{nPPR}}$, acquired magnitude signal in cases of no pre-polarisation field applied. $\alpha ,$ estimated correction to pre-polarisation magnetisation occurring from non-zero ramp time transition between pre-polarisation and evolution fields. β, estimated correction to magnetisation recovery occurring during the evolution to readout ramp and exposure to readout field before detection

^c^Models fit to data acquired at 200 mT. C, correction parameter for Rician noise

^d^Ratio of number of model free parameters compared to the number of data points fitted

**Fig. 2:**
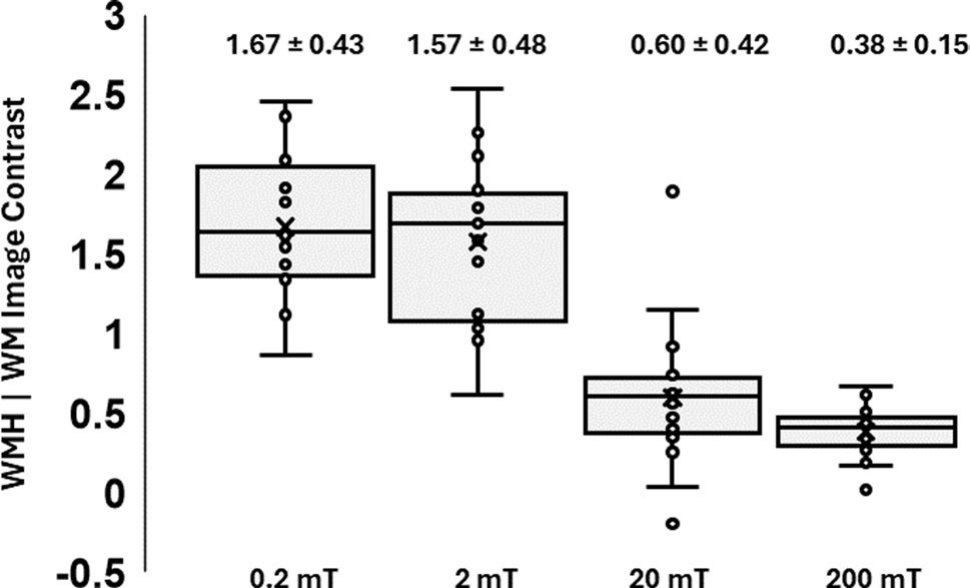
* R*_1_ contrast at each field strength. Box and whisker plots of *R*_1_ contrast between regions of white matter and white matter hyperintensity obtained at 0.2, 2, 20, and 200 mT (*n* = 20, scan visit 1). Each dot represents a single participant. Cohort mean ± standard deviation values are shown

To identify which fitting model was best suited for analysis of brain FCI data, tissue region image contrast and dispersion model adherence were compared. Co-registered 3 T tissue maps were used to extract the *R*_1_ values from WM, GM, and WMH regions and statistical analyses were performed using SPSS (IBM, V29.0.1.0). Performance of fitting models was evaluated for *R*_1_ maps obtained at the lowest field strength of 0.2 mT (*R*_1_^0.2^) because visual inspection confirmed this field had the greatest image contrast between WM and WMH regions (see Fig. 2). First, the image contrast between WMH and WM regions, quantified as the degree of separation between histogram distributions of *R*_1_^0.2^ values ($IC=\frac{{\mu }_{WM}- {\mu }_{WMH}}{{\sigma }_{WM}}$), was compared (*n* = 20, visit 1). Second, the adherence of the calculated *R*_1_^0.2^ values to the dispersion power law model was quantified as mean goodness-of-fit (R^2^) from WMH regions (*n* = 20, visit 1). Significant difference between fitting models was examined using within-subjects ANOVA and post hoc paired *t*-tests with significance level after Bonferroni correction set to 0.01. Models S3 and S4 were expected to improve the model fit due to the inclusion of additional free parameters, but due to potential overfitting, the impact on image contrast and adherence was unknown.

### Tissue contrast and repeatability

For the identified best *R*_1_ mapping approach, repeatability of mean *R*_1_^0.2^ values and dispersion slope, b, extracted from regions of WM, GM, and WMH, were examined by the 95% limits of agreement using the Bland–Altman method and intraclass correlation coefficient (ICC), for a two-way mixed model of absolute values for single measures (*n* = 19, visits 1 and 2). Paired *t*-tests were performed to determine whether there was no significant bias between visit 1 and visit 2 for tissue averaged values of *R*_1_^0.2^ and dispersion slope. The magnitude of Cohen’s d effect size was evaluated between tissue averaged values to examine the extent of *R*_1_^0.2^ and dispersion slope image contrast between different tissue regions using Paired *t*-test (*n* = 20, visit 1). In this analysis, larger values of Cohen’s d indicate larger differences between the tissue groups compared to the extent of variability across each tissue group. Cohen’s *d* values greater than 0.5 and 0.8 are commonly considered to reflect medium and large effect sizes, respectively. One case of visit 2 data was excluded from the repeatability analysis due to failure of the automated 3 T MRI preprocessing pipeline used to generate tissue label maps.

## Results

**Fig. 3:**
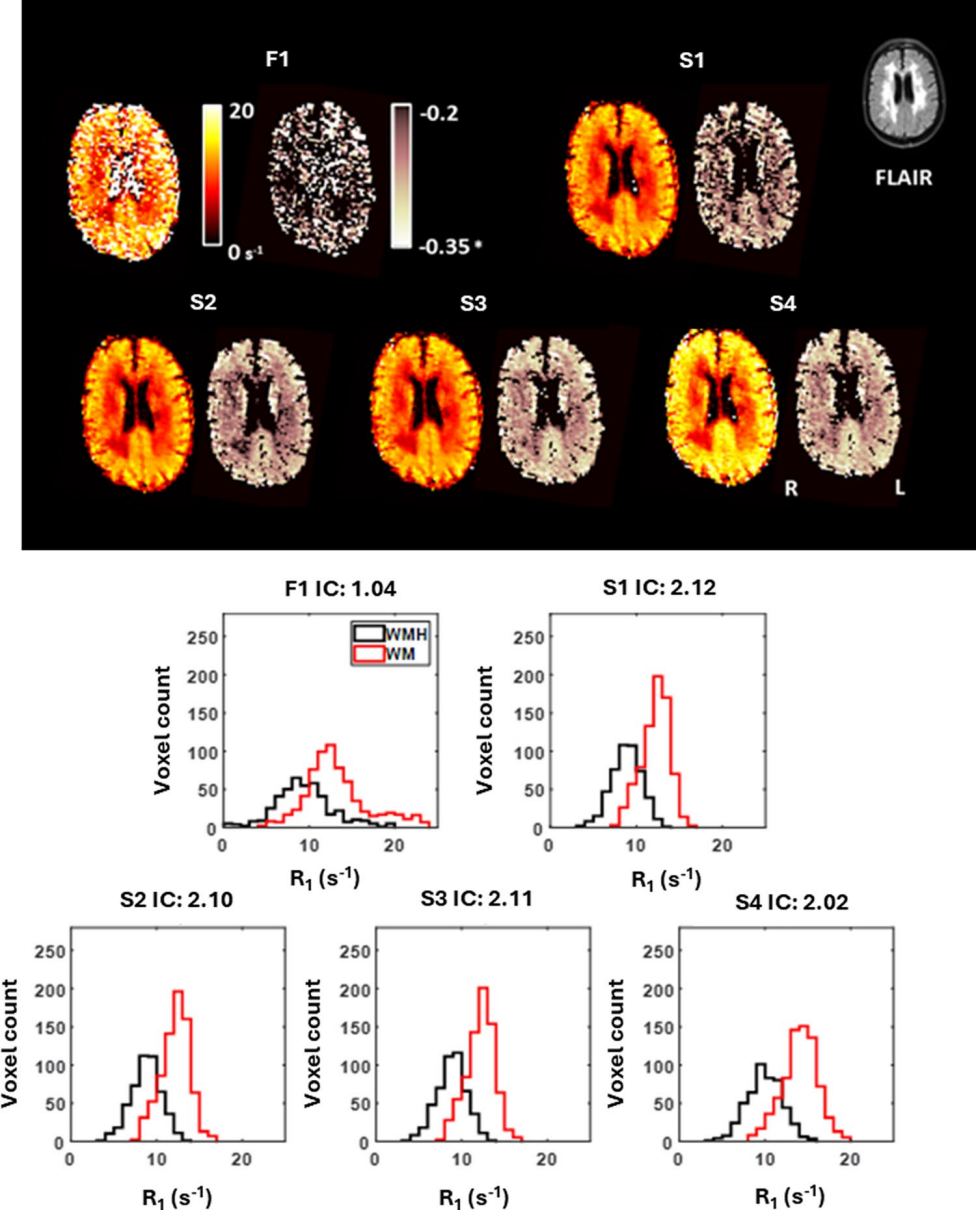
* R*_1_ mapping results for a single participant. Top right of figure shows the FLAIR image obtained from 3 T MRI and co-registered to FCI image space. Brain maps consist of quantitative maps of *R*_1_ at 0.2 mT (left) and dispersion slope b (right). Maps are shown for each fitting model F1 and S1–S4, with motion correction and denoising applied before fitting. Image contrast contained within *R*_1_ maps can be seen to differentiate between SVD regions (hypointense) and WM and GM regions (hyperintense). Matching histogram distributions of *R*_1_ at 0.2 mT are shown for regions of WMH (black) and WM (red)

**Fig. 4:**
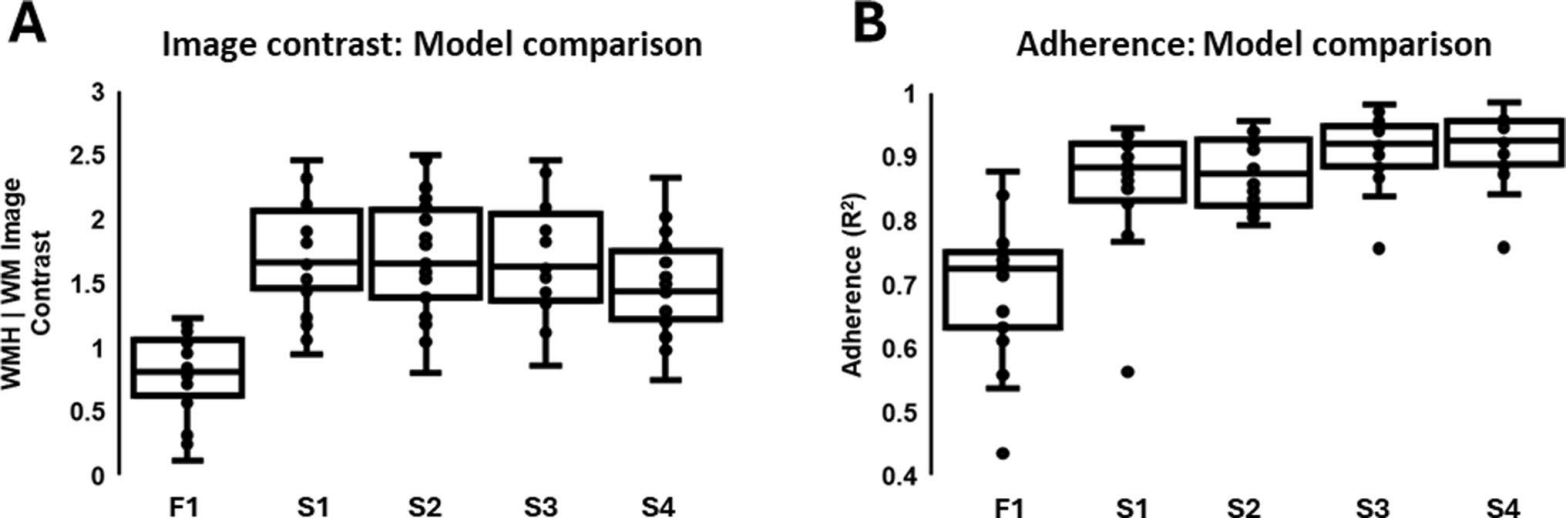
Comparison of image contrast and dispersion power law model adherence. **A** Box and whisker plots of image contrast between white matter and white matter hyperintensity regions for *R*_1_ obtained at 0.2 mT (scan 1). **B** dispersion power law model adherence quantified as goodness-of-fit (R^2^). Each point represents a single participant

Visual inspection of the *R*_1_ maps obtained at 0.2 mT, the dispersion slope maps, and extracted histogram distributions show discernible variations in image quality and image contrast between WM and WMH regions for each preprocessing approach (see Fig. 3). Qualitative appreciation of the *R*_1_ and dispersion slope maps shows that individual fitting of data acquired from each evolution field is not appropriate due to the degree of error propagation. Image contrast between WM and WMH regions was significantly greater for models S1–S3 compared to F1 and S4 (see Fig. 4A). Adherence to the dispersion power law model was observed to be significantly greater (*P* < 0.05) for S3 and S4 models compared to the F1, S1, and S2 models (see Fig. 4B). Hence, fitting model S3 was determined to be the best overall analysis approach.

**Fig. 5:**
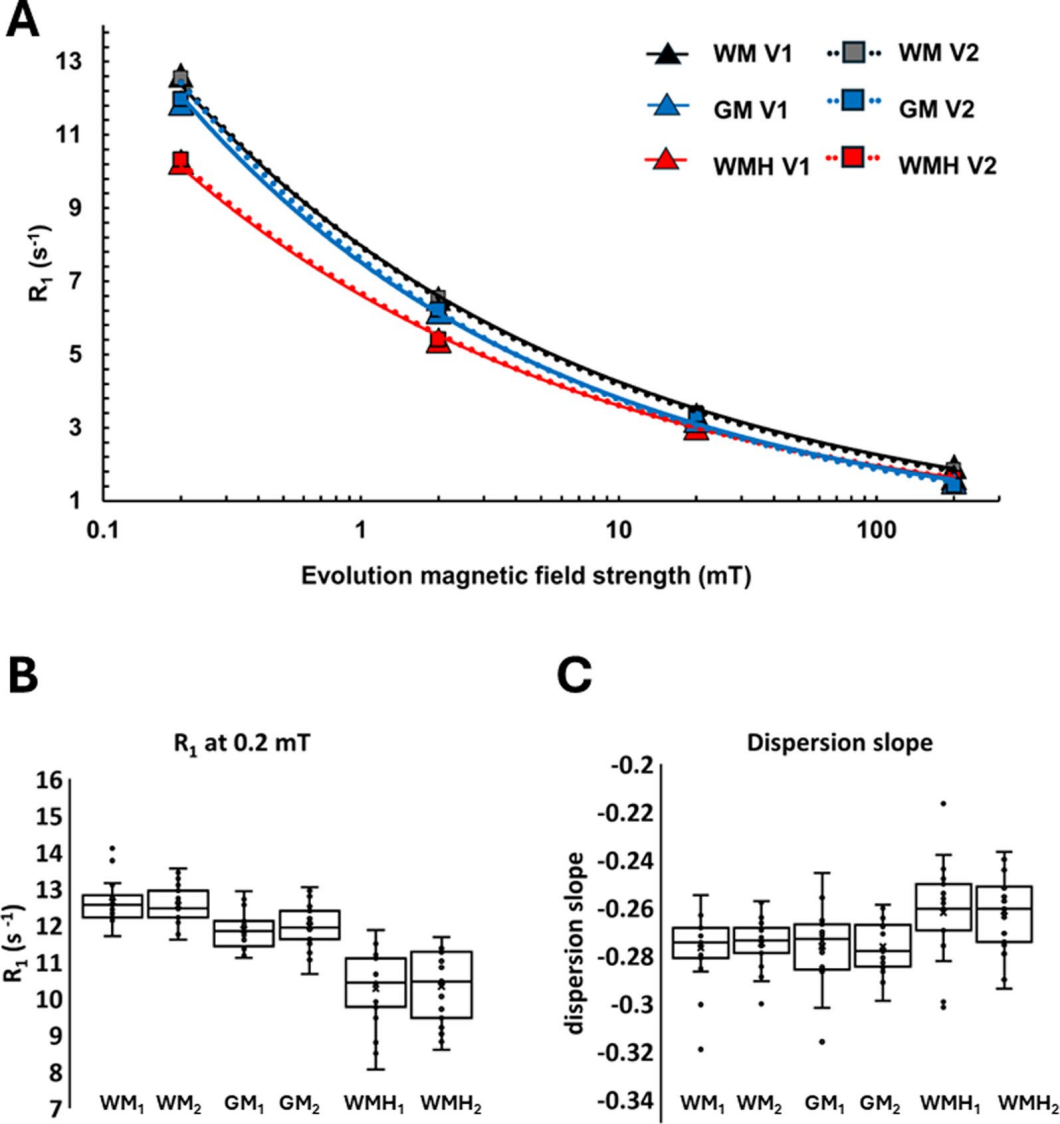
Cohort *R*_1_ mapping results. **A** dispersion of tissue averaged *R*_1_ values with magnetic field strength for white matter (WM), grey matter (GM), and white matter hyperintensity (WMH) regions. For each visit, each point represents the cohort average, triangle marker is visit 1, and square marker is visit 2. **B** box and whisker distribution of averaged *R*_1_ values obtained from WM, GM, and WMH regions for visit 1 and visit 2 scans. **C** Box and whisker distribution of dispersion slope values obtained from WM, GM, and WMH regions for visit 1 and visit 2 scans. Each data point represents a single case

Using the analysis pipeline consisting of motion correction, denoising, and *R*_1_ mapping using model S3, there was no significant difference in bias observed between mean *R*_1_^0.2^ values or dispersion slope values between repeated visits for WM, WMH, and GM (see Table 2 and Fig. 5). For assessment of image contrast, evaluation of Cohen’s d effect size indicated a minimum sample size of 1 was needed to achieve a power of 80% and significance of 5% to detect the effect size of -3.07 observed in this study between mean *R*_1_^0.2^ values extracted from WM and WMH. Similarly, a minimum sample size of 7 was determined for the effect size of 1.48 observed between mean dispersion slope values.

**Table 2:** Repeatability of image contrast and paired t-test effect size

	R_1_ at 0.2 mT			Dispersion slope		
	WM	GM	WMH	WM	GM	WMH
**Repeatability, n = 19**						
Visit 1 Mean ± SD^a^ Visit 2 Mean ± SD	12.6 ± 0.58 12.5 ± 0.54	11.9 ± 0.53 12.0 ± 0.62	10.3 ± 1.04 10.3 ± 0.98	-0.277 ± 0.014 -0.275 ± 0.011	-0.276 ± 0.016 -0.276 ± 0.011	-0.262 ± 0.020 -0.262 ± 0.016
Bias (Paired Sample t-test)^b^	t = 0.547 p = 0.591	t = -0.633 p = 0.534	t = -0.313 p = 0.758	t = -0.544 p = 0.593	t = 0.008 p = 0.994	t = -0.108 p = 0.915
ICC (Absolute, two-way mixed)^c^	0.11, -0.38 – 0.53	0.32, -0.15 – 0.67	0.61, 0.21 – 0.83*	0.17, -0.31 – 0.58	0.08, -0.41 – 0.52	0.32, -0.17 – 0.67
95% limits of agreement (s^−1^)^d^	-1.56 – 1.37	-1.23 – 1.42	-1.71 – 1.85	-0.030 – 0.034	-0.036 – 0.036	-0.041 – 0.042
**Effect size, n = 20**						
Visit 1 Mean ± SD^e^	12.6 ± 0.57	11.8 ± 0.58	10.2 ± 1.04	-0.277 ± 0.014	-0.275 ± 0.016	-0.261 ± 0.020
Cohen’s d Effect Size Vs. WMH^f^	-3.07	-2.06	-	1.48	1.27	-
Minimum Sample Size Vs. WMH (80%, 0.05)^g^	1	5	-	7	8	-

^a^Visit 1 and visit 2 cohort mean ± standard deviation of _R1_ obtained at evolution field of 0.2 mT and dispersion slope, for averaged values extracted from regions of white matter (WM), grey matter (GM), and white matter hyperintensity (WM)

^b^Test of bias between repeated visits

^c^Intraclass correlation results between repeated visits, and 95% confidence interval

^d^Bland–Altman plot 95% limits of agreement

^e^Visit 1 cohort mean ± standard deviation of averaged tissue values

^f^Estimated Cohen’s d effect size for paired t-test between averaged values extracted from WMH, and grey and white matter (GM and WM)

^g^Estimated minimum sample size needed for power of 80% and level of significance of 0.05

**Fig. 6:**
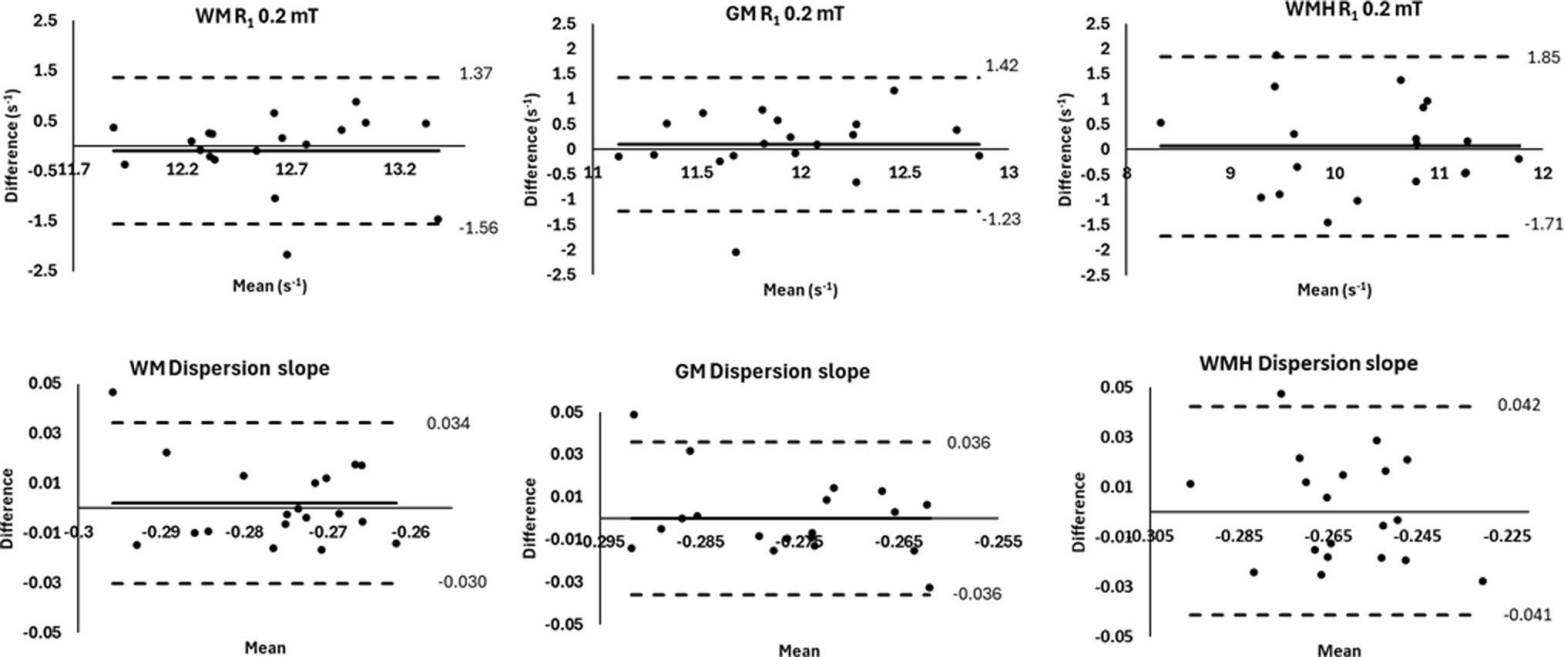
Bland–Altman plots of *R*_1_ dispersion parameters. First row, Bland–Altman plots for tissue averaged *R*_1_ values obtained at 0.2 mT for regions of white matter (WM), grey matter (GM), and white matter hyperintensities (WMH), between visit 1 and visit 2. Second row, dispersion slope, **b** Average line is plotted as solid line, and the limits of agreement are plotted as dashed lines

The limits of agreement between repeated visit measures of mean *R*_1_^0.2^ and dispersion slope values from regions of WM, WMH, and GM are shown in Table 2, and Bland–Altman plots are presented in Fig. 6. Non-significant ICC was observed for regions of WM and GM, indicating no discernible intersubject variation. A significant (*p* < 0.05) ICC of 0.61 (0.21–0.83) obtained for mean *R*_1_^0.2^ values extracted from WMH regions suggests that detectable intersubject variation could be discerned from intrasubject measurement variation.

## Discussion

A repeatability cohort of 20 participants with moderate and severe small vessel disease was used to investigate different analysis approaches and examine the repeatability of *R*_1_ dispersion measurements obtained from FCI in brain. A simultaneous fitting model approach, which took into account sequence inefficiencies and noise, yielded the best overall image contrast and dispersion model adherence. The *R*_1_ dispersion contrast observed between tissue types of white matter, grey matter, and white matter hyperintensities was repeatable at the cohort level.

In addition to inclusion of motion correction and denoising, the robustness of *R*_1_ mapping was improved by selection of the fitting model to maximise the *R*_1_ tissue contrast and the dispersion model adherence of tissue averaged measurements. The validity of fitting approaches that fit data from all field strengths simultaneously was shown compared to fitting data acquired from each field separately. Improvements to image contrast and adherence to the *R*_1_ dispersion power law model were further found to be dependent on the choice of fitting model. Future studies must consider the importance of fitting model selection in respect to the total number of free model parameters, number of data points available, and the desired degree of sensitivity, specificity, and repeatability of outcome measures. Further increases to model complexity that account for specific FCI sequence timings [11], or separate multiexponential tissue components [19, 20], may also yield additional insight into in vivo *R*_1_ dispersion.

To be viable for different brain imaging applications, quantitative imaging of *R*_1_ must yield sufficient sensitivity to discriminate between different tissue states relative to measurement error [21]. In this study, a significant difference in averaged *R*_1_ dispersion measurements between regions of WMH compared to white and grey matter was observed. The contrast in *R*_1_ dispersion was accurately reproduced between the repeated scans, with no significant bias detected. The higher effect sizes observed at lower magnetic field strengths compared to higher fields corroborate previous results in stroke [9], and the feasibility of FCI to interrogate in the dispersion of quantitative *R*_1_ measures below 0.2 T in humans. The repeatability and effect size of multi-field *R*_1_ measurements in brain obtained from FCI in vivo were investigated for the first time and will be used to guide interpretation of longitudinal stroke data sets.

Significant intersubject variation was observed for average 0.2 mT *R*_1_ values extracted from WMH regions, suggesting a degree of sensitivity to underlying differences in tissue state across the cohort. At high field, previous studies have demonstrated a relationship between SVD progression and quantitative MRI measures extracted from both WMH tissue and adjacent tissue [22]. FLAIR imaging performed at 64 mT has also shown potential for identifying cases of moderate and severe WMHs using a portable MRI system [23]. Future work is needed to investigate the utility of FCI measurements of *R*_1_ dispersion contrast in SVD using control populations. Future studies will also be required to examine the extent to which FCI measurements of *R*_1_ at different low field strengths can be reproduced using different classes of fixed and rampable low-field MRI systems [24].

The *R*_1_ dispersion power law relationship observed in white matter agrees with results previously reported for ex vivo tissue [5]. The observable *R*_1_ contrast between white and grey matter was, however, less than previously reported ex vivo [5]. In addition to differences between ex vivo and in vivo states, the extent of partial volume error in this study likely resulted in loss of contrast between tissue types. Partial volume effects were considerable and arose from presence of grey, white matter, and cerebrospinal fluid within single voxel volumes, up to 96 mm^3^ in size [25]. Since partial volume effects present a major challenge for accurate relaxation time mapping at low field [26], improved FCI hardware capabilities are needed to achieve smaller voxel resolutions and improve further the efficacy of *R*_1_ dispersion profiling in vivo.

This study was limited to cases of moderate and severe small vessel disease. Cases of less severe disease without confluent lesions were not included due to hardware constraints on voxel size, single slice acquisition, and feasible scan time. The repeatability and effect sizes reported in this study therefore cannot be generalised to diseases of smaller volume changes owing to the limitations of the voxel size that can be acquired using the mark II FCI scanner. The repeatability results reported were also constrained by the acquisition of a single FCI image slice and scan-to-scan variation in the positioning of this image slice. In future, investigation of early disease changes and smaller pathologies will require improved FCI hardware capability to more efficiently acquire images across a larger number of field strengths and whole brain volumes with finer image resolution and multiple image slices [27].

In conclusion, differences in field dependence of *R*_1_ could repeatably be discerned between normal and abnormal brain tissue arising from moderate and severe cerebral small vessel disease. The effect size to differentiate between normal and abnormal tissue was improved by tailoring the model fitting approach used.

## Supplementary Information

Below is the link to the electronic supplementary material.

Supplementary file1 (DOCX 584 KB)

Supplementary file2 (PNG 359 KB)

Supplementary file3 (PNG 29 KB)

Supplementary file4 (PNG 151 KB)

## Data Availability

The data that support the findings of this study are not openly available due to reasons of sensitivity and are available from the corresponding author upon reasonable request.
