# Supplementary material for: Field-cycling imaging yields repeatable brain R1 dispersion measurement at fields strengths below 0.2 Tesla with optimal fitting routine
Source: MAGMA. 2025 Feb 15;38(3):465–74. doi: 10.1007/s10334-025-01230-w (PMC12255585; doi:10.1007/s10334-025-01230-w)
Supplement: Supplementary file 1 — Supplementary file1 (DOCX 584 KB) [file 10334_2025_1230_MOESM1_ESM.docx]

**Field-Cycling Imaging yields repeatable multi-field R_1_ measurements in brain at field strengths below 0.2 Tesla**

**Supplementary Materials**

**Content**

1. Comparison of denoising approaches
2. Motion correction findings
3. Supplementary figures
4. References

**1. Comparison of denoising approaches**

This work was undertaken to examine the efficacy of different denoising approaches for Field-Cycling Imaging data acquired in brain. The best denoising approach was determined by comparing image contrast between regions of white matter and white matter hyperintensities and adherence to the dispersion model by goodness-of-fit R^2^. Here the results of comparing different denoising approaches are reported for the fitting model S3.

Methods: After motion correction, images were denoised using either a median filter with square kernel sizes of length 2, 3, or 4 voxels (Median_2-4_), a pretrained denoising convolutional neural network (dnCNN) approach contained within MATLAB (MathWorks, USA), introduced in R2017b ^1^, or block-matching and 3D filtering approach (BM3D, Tampere University, Finland) ^2^, with noise standard deviation set to either 100% (BM3D) or 120% (BM3D_1.2_) for each case. Significant difference between denoising methods was examined using within-subjects ANOVA and post-hoc paired t-tests with significance level set after Bonferroni correction to 8.3E-3.

Results: Overall, the denoising convolution neural network (dnCNN) approach was determined to be the best overall preprocessing approach by comparison of dispersion power law adherence and image contrast.

Visual inspection of the R_1_ maps obtained at 0.2 mT, dispersion slope maps, and extracted histogram distributions show discernible variations in image quality and image contrast between WM and WMH regions for each denoising approach (see Figure A1). Qualitative appreciation of the images shows that the use median filter with kernel size greater than 2 × 2 voxels was found to over smooth the image data and it was concluded that this method was inappropriate for the acquired image resolution in this study and was excluded from subsequent analysis. Above this kernel size threshold, substantial partial volume error was observed as artificially high R_1_^0.2^ values in regions of cerebral spinal fluid.

According to figure A2, use of denoising improved dispersion model adherence and image contrast. DnCNN denoising yielded significantly (P < 0.001) greater R_1_ contrast between regions of WM and WMH (cohort mean ± std: 1.67 ± 0.43) compared to BM3D (1.28 ± 0.40), BM3D_1.2_ (1.53 ± 0.44), and Median filter with kernel size of 2-by-2 voxels (1.38 ± 0.46).

Compared to fitting of data with no motion correction and no denoising, DnCNN denoising improved R_1_ mapping goodness-of-fit overall by 12.3% (WM), 16.3% (GM), and 14.4% (WMH), (see Figure A3A). Differences in bias between denoising approaches for averaged parameter values were detectable for each tissue region after Bonferroni correction (see Figure A3B and C). Compared to the DnCNN denoising approach, the percentage difference of the cohort averaged WMH R_1_^0.2^ and dispersion slope was below 5% (range: 0.8 – 4.6 %) for the different preprocessing approaches.

Discussion: R_1_ mapping results were dependent on the denoising approach used. The pretrained denoising convolutional neural network (“DnCNN”) approach contained within MATLAB, introduced in R2017b ^1^, yielded significantly increased R_1_ contrast compared to a median filter (kernel size of 2-by-2 voxels) and BM3D ^2^. The performance of BM3D may have been limited for this imaging data due to the high levels of noise ^3^. Visually, DnCNN and BM3D yielded improved image quality compared to the median filters, where larger kernel sizes resulted in substantial image blurring and compounded partial volume errors. Because of the limited image resolution acquired, the results of this study may however have limited generalisability to small volumes of grey matter and brain pathology such as punctate foci of WMHs ^4,5^. In addition, the bias detected below 5% between R_1_ values obtained from the different denoising approaches warrants further investigation ^6^. This will require comparison to imaging data acquired with multiple signal averages and therefore improvements to imaging hardware are needed to facilitate feasible scan times. Future collaborative research efforts are needed to utilise the power of disease specific data-sets to develop tailored denoising solutions for low-field and translate approaches proven at high-field ^7-9,3^.

**2. Motion correction findings**

A total scan time of 30 minutes was needed to acquire the 20 field-cycling imaging data sets. Because of this length of scan time, we deployed motion correction to examine and address potential head movement across FCI data sets. Whilst motion correction is typically deployed to resolve images across whole 3D volumes of brain, we were limited to correcting yaw rotation and 2D displacement since only a single image slice was acquired. Motion correction was implemented and the extent of motion of measured before and after correction was examined.

*Methods:* Motion correction was performed using a rigid-body spatial transformation to the mean FCI image using SPM12. Reduction in head motion was quantified for in-plane displacements and yaw rotation ^10^.

*Results:* Motion correction led to a measurable reduction of mean x, y, and yaw (rotation) motion by 93.1% (cohort mean ± std: 0.73 ± 0.26 vs. 0.05 ± 0.02 mm), 93.8% (0.84 ± 0.44 vs. 0.05 ± 0.02 mm), and 93.2% (5.9 ± 3.4 vs. 0.4 ± 0.1 millirad), respectively. Compared to original data, motion correction alone improved R_1_ mapping goodness-of-fit (R^2^) overall by 2.2% (WM), 3.3% (GM), and 2.2% (WMH) (see Figure A3A).

*Discussion:* Head motion between images acquired from FCI was detected in this study and was comparable to the magnitude of motion reported from other MRI studies performed at high-field with similar scan length ^10^. The implemented rigid body correction approach was found to be effective for realignment of single-slice brain images and resulted in detectable improvements to model goodness-of-fit. Head motion may be more severe in other patient cohorts, including stroke, and potential biases introduced to R_1_ mapping results and performance of different correction approaches should be considered ^11^.

**3. Supplementary figures**


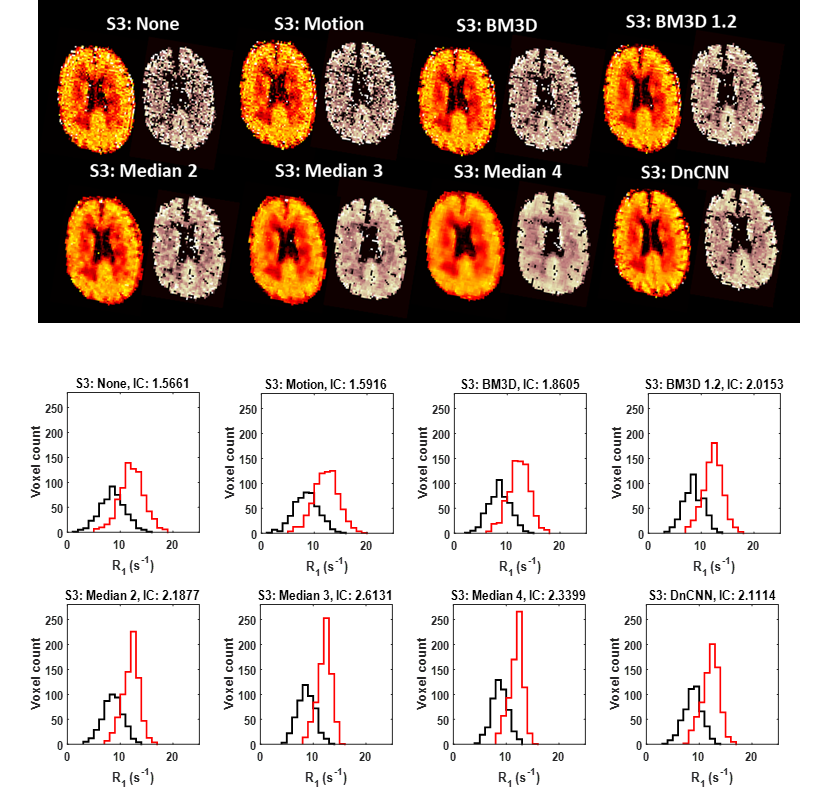


**Figure A1. R_1_ mapping results for a single participant.**

Brain maps consist of quantitative maps of R_1_ at 0.2 mT (left) and dispersion slope b (right). Maps shown were analysed using fitting model S3, and motion correction was applied before each denoising approach. Matching histogram distributions of R_1_ at 0.2 mT are shown for regions of WMH (black) and WM (red).


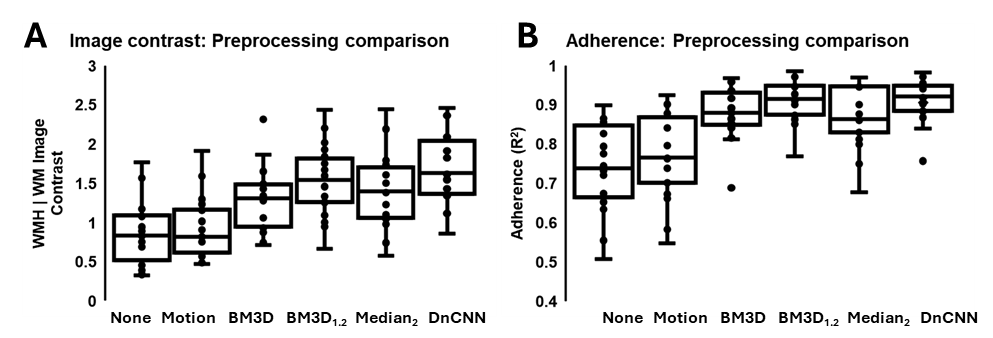


**Figure A2. Comparison of image contrast and dispersion power law model adherence**

Figure A2A, box, and whisker plots of image contrast between white matter and white matter hyperintensity regions for R_1_ obtained at 0.2 mT (scan 1). Figure A2B, dispersion power law model adherence quantified as goodness-of-fit (R^2^). Each point represents a single participant.


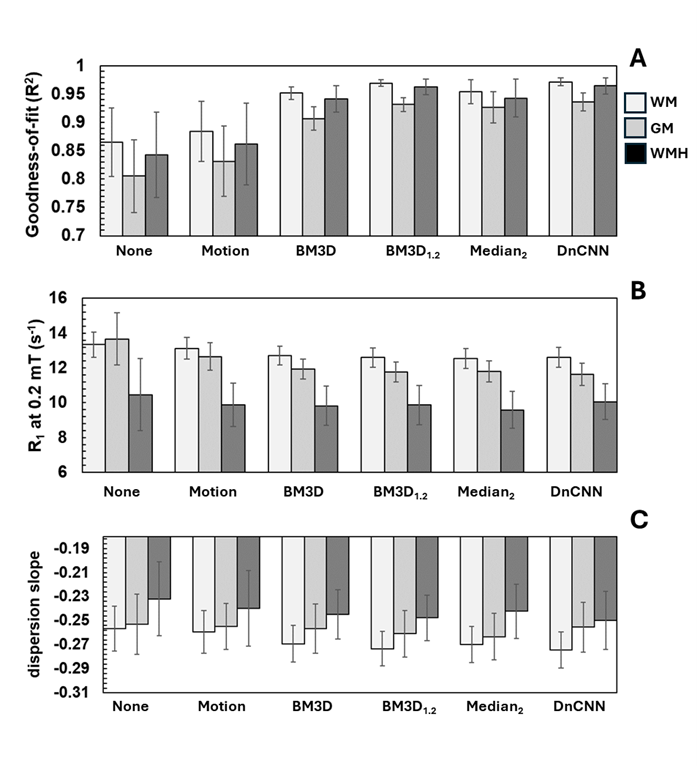


**Figure S3. Comparison of preprocessing methods**

Figure A3A, the cohort average R_1_ mapping goodness-of-fit for each tissue region is shown for 1) white matter (white), 2) grey matter (grey) and 3) white matter hyperintensities (dark grey) for each denoising approach. Error bars represent the cohort standard deviation. Figure A3B, comparison of cohort averaged R_1_ at 0.2 mT. Figure A3C, comparison of cohort averaged dispersion slope. Data fitted with model S3.

**4. References**

1. Zhang K, Zuo W, Chen Y, Meng D, Zhang L. Beyond a gaussian denoiser: Residual learning of deep CNN for image denoising. *TIP*. 2017;26(7):3142–3155. doi: 10.1109/TIP.2017.2662206.

2. Dabov K, Foi A, Katkovnik V, Egiazarian K. Image denoising by sparse 3-D transform-domain collaborative filtering. *TIP*. 2007;16(8):2080–2095. doi: 10.1109/TIP.2007.901238.

3. Fan L, Zhang F, Fan H, Zhang C. Brief review of image denoising techniques. *Vis Comput Ind Biomed Art*. 2019;2(1):7–12. doi: 10.1186/s42492-019-0016-7.

4. Kim KW, MacFall JR, Payne ME. Classification of white matter lesions on magnetic resonance imaging in elderly persons. *Biological psychiatry (1969)*. 2008;64(4):273–280. doi: 10.1016/j.biopsych.2008.03.024.

5. Wardlaw JM, Smith C, Dichgans M. Small vessel disease: Mechanisms and clinical implications. *The Lancet Neurology*. 2019;18(7):684–696. doi: 10.1016/S1474-4422(19)30079-1.

6. Kay K. The risk of bias in denoising methods: Examples from neuroimaging. *PloS one*. 2022;17(7):e0270895. doi: 10.1371/journal.pone.0270895.

7. Maier O, Schoormans J, Schloegl M, et al. Rapid T1 quantification from high resolution 3D data with model‐based reconstruction. *Magnetic resonance in medicine*. 2019;81(3):2072–2089. doi: 10.1002/mrm.27502.

8. Maggioni M, Katkovnik V, Egiazarian K, Foi A. Nonlocal transform-domain filter for volumetric data denoising and reconstruction. *TIP*. 2013;22(1):119–133. doi: 10.1109/TIP.2012.2210725.

9. Mansour Y, Heckel R. Zero-shot Noise2Noise: Efficient image denoising without any data. *CVPR*. Jun 2023:14018–14027.

10. de Vries CF, Staff RT, Waiter GD, Sokunbi MO, Sandu AL, Murray AD. Motion during acquisition is associated with fMRI brain entropy. *JBHI*. 2020;24(2):586–593. doi: 10.1109/JBHI.2019.2907189.

11. Marques JP, Simonis FFJ, Webb AG. Low‐field MRI: An MR physics perspective. *Journal of magnetic resonance imaging*. 2019;49(6):1528–1542. doi: 10.1002/jmri.26637.
