# Supplementary figures and images for: Field-cycling imaging yields repeatable brain R1 dispersion measurement at fields strengths below 0.2 Tesla with optimal fitting routine
Source: MAGMA. 2025 Feb 15;38(3):465–74. doi: 10.1007/s10334-025-01230-w (PMC12255585; doi:10.1007/s10334-025-01230-w)

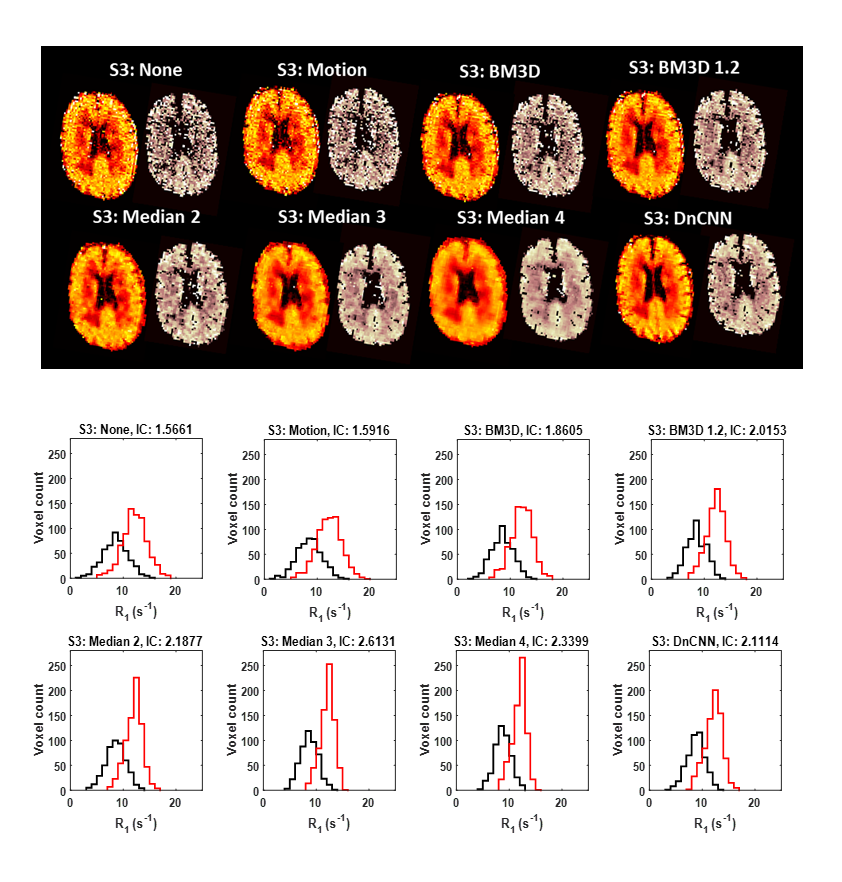

Supplement: Supplementary file 2 — Supplementary file2 (PNG 359 KB) [file 10334_2025_1230_MOESM2_ESM.png]

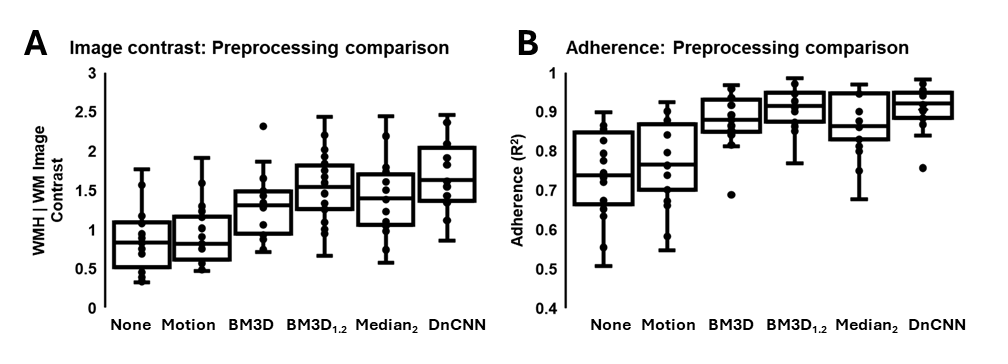

Supplement: Supplementary file 3 — Supplementary file3 (PNG 29 KB) [file 10334_2025_1230_MOESM3_ESM.png]

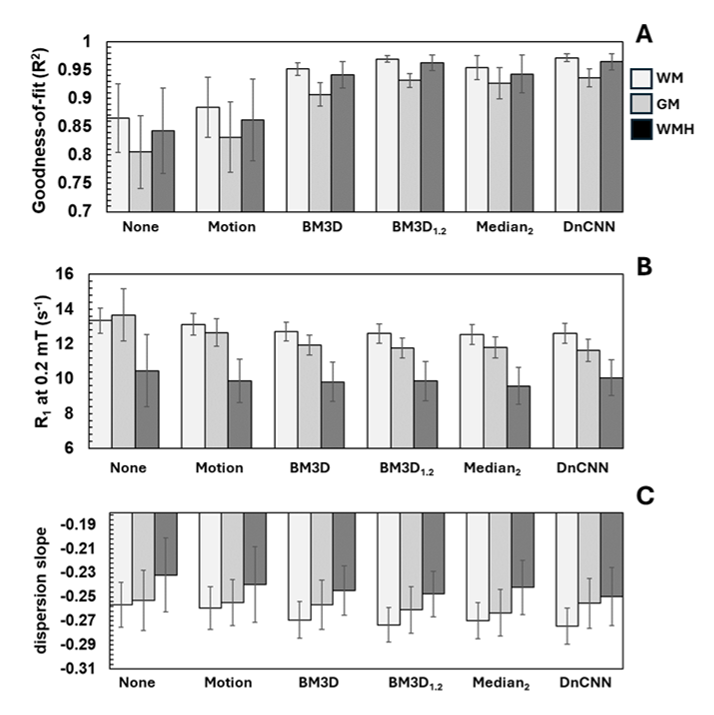

Supplement: Supplementary file 4 — Supplementary file4 (PNG 151 KB) [file 10334_2025_1230_MOESM4_ESM.png]
